# Monitor displays in radiology: Part 2

**DOI:** 10.4103/0971-3026.50819

**Published:** 2009-05

**Authors:** IK Indrajit, BS Verma

**Affiliations:** Department of Radiodiagnosis and Imaging, Command Hospital (Air Force), Bangalore-560 007, India; 1 Department of Radiodiagnosis and Imaging, Base Hospital, Lucknow - 226 002, India

**Keywords:** Brightness, color bit depth, grayscale display function, luminance, medical-grade displays, performance parameters, refresh and response rates, resolution, task group-18 tests, viewing angle

## Abstract

Monitor displays play an important role in modern radiology practice. Practicing radiologists need to be familiar with the various performance parameters of medical-grade displays. A certain amount of technical knowledge is useful when making purchasing decisions since the right choice of equipment can have a great impact on the accuracy, efficiency, and speed in the radiology department.

## Introduction

The fundamental concepts regarding monitor displays were dealt with in the first part of this article, where we discussed the need for possessing a basic knowledge of monitors and the principal differences between cathode ray tube (CRT) and liquid crystal display (LCD) monitor technologies. We also gave simplified definitions of terms in monitor technology.

Briefly, two different monitor display technologies have emerged in the computer industry, i.e., CRT and LCD technologies. Broadly speaking, CRT technology is a mature technology, while LCDs are a recent innovation. In LCD monitors, light generation and light modulation are physically separated, unlike in CRT technology. CRT monitors are bulky and heavy and take up a lot of space on the desk. LCD monitors, on the other hand, are thinner and lighter and have a small footprint. LCD technology is based on the fact that liquid crystals can transmit and change polarized light under the effect of an electric current. An LCD monitor is a multilayered light valve ‘sandwich.’ It has an LCD panel that contains liquid crystal material which forms tiny color pixels, thereby creating an image on screen.

In this, the second part of the article, we focus on different performance parameters and a few quality issues that radiologists should be aware of.

## Performance parameters in monitor display

Monitor display have a few important performance parameters. In this section we discuss the terminology and some of the basic concepts.

### Screen size:

**Figure 1 (A,B):**
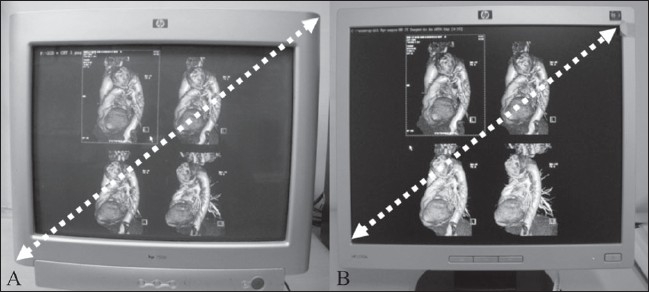
Screen sizes. In CRT monitors (A), the screen size is measured diagonally from the outside edges of the display casing. In LCD monitors (B), the screen size is measured diagonally from the inside of the beveled edges. Thus a 17-inch LCD display is comparable to a 19-inch CRT display. The CRT display includes 2 inches of casing, as well

The screen size is measured diagonally, from ‘corner to corner.’ Common monitor sizes, in inches, are 15″, 17″, 19″, and 21″. In the case of CRT monitors, the diagonal measurement is taken from the outside edges of the display casing and, therefore, the active display area is actually a few inches lesser. In LCD monitors, the diagonal measurement is taken from the inside of the beveled edge. As a result, a 17″ LCD display is comparable to a 19″ CRT display[1] [Figure 1].

### Aspect ratio:

This is the ratio of the width of a screen image to its height. The aspect ratio for most CRT monitors is 4:3. However, the trend today is in favor of flat screens and wide-screen LCD monitors with aspect ratios of 16:9 and, occasionally, even 16:10 or 15:9, are common today.[2]

### Landscape *vs* portrait:

**Figure 2 (A,B):**
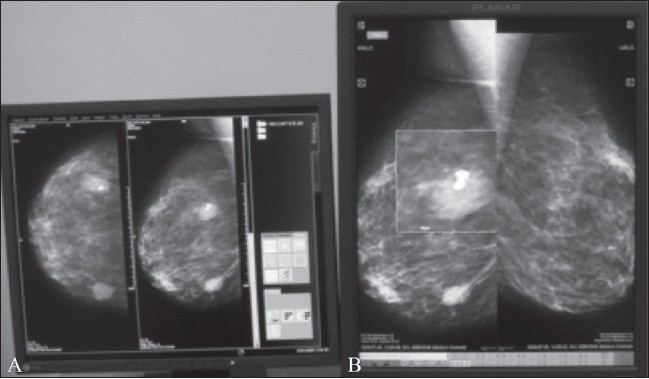
Landscape (A) versus portrait (B) mode. By virtue of being lightweight and thin, LCD monitors can be placed in a portrait mode that corresponds with the 17×14 format of images obtained in chest and skeletal radiography. Mammography images can be optimally viewed in the ‘portrait’ (B) rather than the ‘landscape’ mode (A), as shown here

Until recently, images in radiology were viewed in the landscape mode, a tradition set by the computer industry. This mode is clearly not optimal for use in diagnostic imaging.[3] LCD monitors, being lightweight and thin, can be easily turned around and placed in a portrait mode, which corresponds with the image format of radiology images. A 17 × 14 image is optimally viewed in the ‘portrait’ rather than ‘landscape’ mode. The ‘portrait’ mode is particularly useful for chest and skeletal radiography and for mammography [Figure 2].[4]

### Pixel and dot pitch:

Pixel or ‘picture element’ is the basic unit used for generating a video or computer picture and is measured in millimeters. In the CRT display screen, a pixel is the distance between phosphor dots, whereas in the LCD display screen it is the distance between cells of the same color. A dot pitch is essentially a dot with a given color and brightness value. A smaller dot pitch implies a sharper image, as there are more dots in an area of a given size. In computer monitors, the pixel numbers are dependent on the aspect ratio of the screen (its horizontal size compared to its vertical size) and the display size. Standard monitors with a 4:3 aspect ratio have a width of 1024 pixels and a height of 768 pixels. Widescreen display monitors with a 16:9 aspect ratio have a width of 1024 pixels and a height of 576 pixels.[1]

### Resolution:

The resolution is calculated as the product of the number of pixels in the horizontal axis (rows) and the number in the vertical axis (columns) on a computer screen. Commonly available computer monitors have resolutions of 640 × 480, 800 × 600, or 1024 × 768.[3]

In a CRT monitor, the horizontal resolution is limited by bandwidth and spot size, while the vertical resolution is limited by line spacing and spot size.[5] In comparison, the resolution in an LCD monitor is determined by pixel pitch (the product of vertical and horizontal pixels)[5] and ranges commonly from 1 megapixel (MP) to 5 MP. With ongoing refinements in technology, high-resolution monitors with resolutions as high as 9 MP and higher have started becoming available.

In CRT monitors, the resolution can be changed to match the frequency emitted by the video signal. This advantageous feature is referred to as ‘multisync.’[6] LCD monitors, in contrast, have a fixed resolution, which is termed the native resolution.

### Grayscale display function (GSDF) and grayscale range:

In general, the number of available shades of gray in consumer displays is limited to 256 (8 bit). Medical displays need a much wider grayscale range so that they can render nearly every shade of gray; the latest medical monitors offer upto 4096 shades of gray (12 bit). It is well known that color display monitors have a poor grayscale response.[4]

With the availability of a variety of monitors in radiology, there is a need to ensure that different displays show the same image consistently and that too over a long period of time. An optimal grayscale response for monitors can be achieved by calibration of monitors according to the guidelines given in part 14 of the DICOM standard: the grayscale display function (GSDF).[7] The DICOM GSDF ‘specifies a standard relationship for the shades of gray.’[8] It recommends the use of calibration systems for measuring the monitor display's white level, black level, and the shades of gray in between, with allowances for the ambient light. The use of lookup tables (LUTs) converts a particular display to a DICOM-standard display, thereby ensuring consistency.

### Color bit depth:

The color bit depth is the number of bits used to describe the color of a single pixel. The bit depth of monochrome monitors is 2, i.e., it offers two colors. Moving up the scale, color graphics adapter (CGA) and enhanced graphics adapter (EGA) monitors have bit depths of 4 and 8, allowing 4 and 16 possible color combinations, respectively. Video graphics array (VGA) monitors have a bit depth of 16, which permits 256 color combinations. A high-color extended graphics array (XGA) monitor allows 65,536 colors due to its bit depth of 32. A true color super video graphics array (SVGA) has a bit depth of 64, which permits 16,777,216 colors.[9] Knowledge of color bit depth is useful when working with color Doppler as well as CT scan, and MRI images, where color-encoded functional imaging is displayed on color monitors.

### Brightness / luminance:

In monitors, the term brightness refers to the ‘emitted luminous intensity on screen.’[10] It is measured in candela per square meter (cd/m^2^ or nits). A higher cd/m^2^ or nit value indicates a higher onscreen brightness.[10] Luminance is defined as the ‘absolute quantity of radiation emitted from a given source of visible electromagnetic radiation.’[10] Luminance ratio (LR), the ratio of maximum luminance to minimum luminance (L_max_ / L_min_), is also important.

Let us first examine the luminance of viewboxes. Low-intensity viewboxes have luminance values of 1000–2000 cd/m^2^, while the luminance of high-intensity viewboxes is 2000–3000 cd/m^2^. The viewboxes for viewing mammograms have higher luminance, ranging from 3500 to 5000 cd/m^2^.[11] With regard to monitor displays, CRT monitors generally have a maximum phosphor luminance of about 450 cd/m^2^ and an LR of about 900:1. These values are higher in the case of LCD monitors, with a maximum luminance of about 1000 cd/m^2^ and an LR of about 1200:1.[5]

Medical-grade display systems have certain unique features that are essential for achieving accuracy and efficiency in the diagnosis of lesions. Medical displays maintain consistent contrast over a much larger angle and reduce the deleterious effects of ‘noise’ caused by luminance variations and color variations. Medical-grade displays can automatically calibrate themselves, thereby matching the contrast to every level of brightness.

Medical-grade displays are also designed to provide very high luminance. Compared to consumer-grade displays, medical displays have a much higher luminance range: 250–300 cd/m^2^ for consumer-grade displays *vs* more than 1000 cd/m^2^ for medical displays.[12] The higher luminance offered by medical displays results in better image quality, making subtle lesions easier to detect.[12]

### Contrast ratio:

It is defined as the ratio of the white luminance to black luminance. Simply put, it describes the ‘ability to produce bright whites and the dark blacks.’[3] Consumer-grade displays have a contrast ratio that is usually in the range of 300:1; in comparison, medical displays are substantially better, with values of nearly 1000:1.[12]

In the case of LCD displays, monochrome and color displays are available for the diagnostic viewing of medical images. When currently available monochrome and color LCD displays are compared, there are a few important differences. The native brightness and contrast of monochrome displays are higher than those of color LCD displays.[13]

### Viewing angle:

Viewing angle is ‘the maximum angle at which the display can be viewed with acceptable quality.’[14] An optimal viewing angle ensures that the images are clearly defined, with accurate colors, in horizontal and vertical ranges. A larger viewing angle advantageously allows multiple users to review images simultaneously. An incorrect viewing angle may cause the image to dim or disappear, or colors may be misrepresented. More significantly, subtle changes can be missed if the radiologist's position relative to the screen is less than optimal.

**Figure 3 (A-D):**
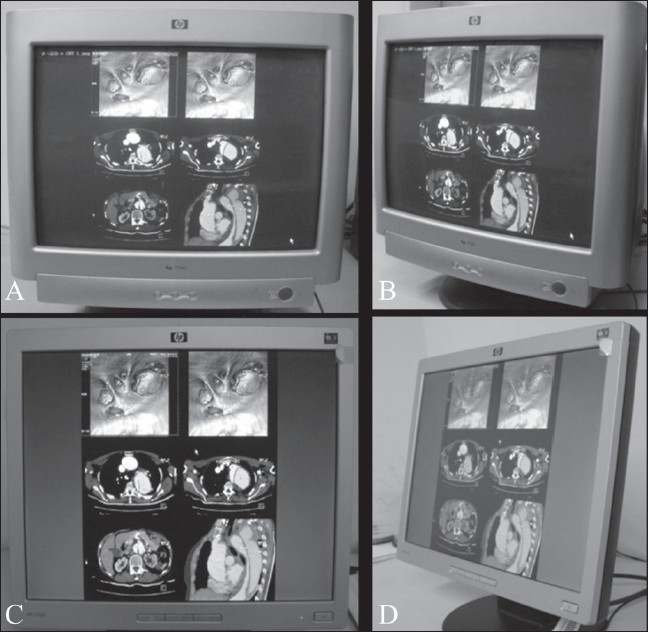
The importance of the viewing angle. A CRT display shown directly in front of the viewer (A) and off-angle (B). An LCD display shown directly in front of the viewer (C) and off-angle (D). The contrast ratio in CRT displays remains constant over a wide viewing angle (B) unlike LCD displays (D) where it drops rapidly. An incorrect viewing angle for LCDs may make images dim or disappear or misrepresented with off-axis distortion, all of which can result in the missing of subtle lesions. A CRT monitor has a larger viewing angle that advantageously allows multiple users to review images simultaneously

The contrast ratio in CRT displays remains constant over a wide viewing angle.[4] In comparison, the contrast ratio in LCD displays drops rapidly from the maximum to about 10:1 for a viewing angle of 85°[4] [Figure 3]. It is therefore recommended that the person viewing an image on a monitor should be seated directly, perpendicularly in front of the monitor; a viewing angle close to 90° eliminates ‘off-angle’ viewing. On-axis viewing is considered acceptable with both CRT and LCD monitors, but off-axis distortions can occur with LCD monitors.[15]

### Refresh rate:

In a CRT monitor, the electron beam hits phosphor-coated dots, causing pixels of an image to flicker. Individually, each pixel is hit 60–80 times a second.[16] Flicker is annoying to the human visual system.[1] It is avoided in a CRT monitor by an adequate refresh rate, denoted by the number of times a display monitor is illuminated. The refresh rate is measured in Hertz (Hz). A refresh rate of at least 70 Hz is necessary in case of diagnostic CRT monitors.[17] For larger CRT monitors a refresh rate of 85 or even higher may be needed. In case of LCD monitors, there is no flicker due to the slow response time.[1]

### Response rate:

This term is specific for LCD monitors; it describes the amount of time taken for a pixel to change from active (black) to inactive (white).[18] It is measured in milliseconds, with a lower number implying faster transitions and fewer visible artifacts like the ‘ghosting’ effect when the image moves across the screen. In color LCDs, response rate denotes the speed with which the monitor's pixels change colors.

### Touch screen:

With the use of touch-screen technology the operator can navigate by manually touching the surface of a monitor. It is made possible by different technologies such as infrared sensors, electronic capacitors, or pressure-sensitive resistors.

## Quality issues in medical-grade displays

Various regulatory bodies in the computer, health, medical, and radiology industries offer guidelines for maintaining quality in monitor displays. For the practicing radiologist, three amongst them are relevant, namely a) AAPM: TG-18, b) ACR guidelines, and c) DICOM GSDF.

**Figure 4:**
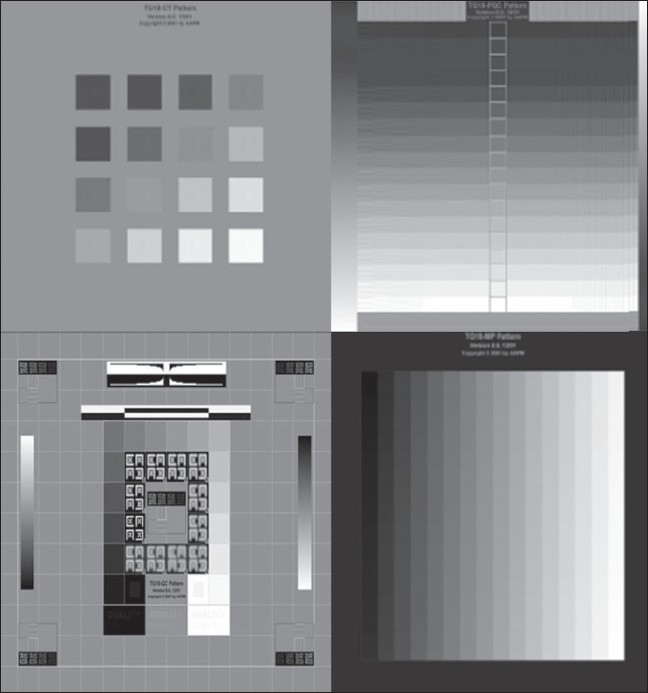
Examples of TG 18 Test Patterns, which facilitate quality control, calibration and consistency across different medical displays. (TG stands for Task Group)

The American Association of Physicists in Medicine (AAPM) has devised comprehensive quality tests for medical displays.[19] These are termed as Task Group (TG)-18 tests. They facilitate quality control, calibration, and the maintenance of consistency of different medical displays [Figure 4].

The American College of Radiology (ACR) recommends that workstation monitors of modalities other than mammography should have a maximum luminance of at least 171 cd/m^2^.[15] The parameters for display capabilities are stricter and more demanding for mammography monitors; ideally, monitors used in mammography should have a maximum luminance of at least 250 cd/m^2^.[15]

## Future

Monitor display manufacturers constantly bring out a slew of changes each year. Among the many innovations that have been introduced are, for example, larger-sized medical monitors,[20,21] wide screens with dual-display systems,[22] the addition of more megapixels (even up to 20 MP),[23] constant integration with the DICOM calibration curve,[24] higher luminance values, better uniformity in brightness and luminance, improved resolution, wider viewing angles, an increase from 8 bits per pixel to 10–14 bits per pixel,[25] touch-screen functions, picture-in-picture, Wi-Fi access, better integration with graphic boards, and seamless support for analogue and digital video.

There are other innovations too, which include self-diagnostics, blue tinting of backlight,[26] embedded ambient light sensors,[27] in-plane switching for overcoming low contrast and narrow viewing angles,[13] color nonuniformity compensation,[28] and user-defined gamma and DICOM LUT presets.[29]

Technological advances are continuing at various levels in the image chain. Consequently, the right and optimal medical-grade display system for radiology practice has not yet been made. This raises the question: ‘Is it possible that we will ever have an ideal monitor and, if so, what would be its specifications?’ There are a few comprehensive white papers that the interested reader could refer to for more detailed information on the basic features and ongoing advances in medical-grade displays.[4,12,13,30–32]

## Conclusion

Monitor displays are an integral part of modern radiology practice. As practicing radiologists, we need to know the technology behind the monitor displays at our workplace. There is a need to be familiar with the various performance parameters of medical-grade displays. Technical knowledge of the ongoing advances in medical-grade displays will be useful when making purchasing decisions and will help improve accuracy, efficiency, and speed in the radiology department. With this end in view, we present this basic review of monitor displays for radiologists.
